# Parental Satisfaction and Associated Factors Toward Their Child's Anesthesia Service at a Comprehensive Specialized Referral Hospital in Ethiopia, 2021: A Cross-Sectional Study

**DOI:** 10.3389/fped.2022.849969

**Published:** 2022-06-06

**Authors:** Biruk Adie Admass, Abebaw Shiferaw Hailemariam, Abatneh Feleke Agegnehu, Amare Belete Getahun

**Affiliations:** Department of Anesthesia, College of Medicine and Health Sciences, University of Gondar, Gondar, Ethiopia

**Keywords:** parents, children, satisfaction, anesthesia service, surgery

## Abstract

**Background:**

Parental satisfaction is a key measure of the quality of a child's anesthetic care. Understanding of parents' opinions and satisfaction about their child's anesthesia service in the hospital is vital for hospital funding and parent experience.

**Objective:**

The purpose of this study was to determine the level of parental satisfaction with their child's anesthetic care and the factors that contributed to that satisfaction.

**Methods:**

An institution-based cross-sectional study was undertaken between March and June 2021. A structured questionnaire with the background characteristics of parents and children was used to collect data. A total of 238 parents were included in the study. To find parameters linked to parental satisfaction with their child's anesthetic service, bi-variable, and multi-variable logistic regression analyses were used. Crude odds ratio and adjusted odds ratio with a 95% confidence interval (*CI*) were estimated. Variables with a *p*-value < 0.05 were considered statistically significant in multivariable analysis.

**Result:**

The proportion of parental satisfaction toward their child's anesthesia service was 77.7% (95% *CI*: 72.3, 82.4%). Non-anxious, male, employed, and urban resident parents and parents of pre-medicated children were associated with high satisfaction scores.

**Conclusion and Recommendation:**

Overall, parents' satisfaction with their child's anesthesia service was promising. Parents who were non-anxious, male, employed, and lived in an urban area and whose child had received sedative premedication had high rates of parental satisfaction with their child's anesthetic care.

Parents from rural areas, as well as worried and female parents, should receive extra care. Preoperative anxiety is reduced when parents are given enough and understandable information. Thus, the provision of comprehensive information on their child's anesthesia care process and psychological or emotional support to parents are necessary to boost their satisfaction.

## Introduction

Patient satisfaction is a complicated notion that is based on a patient's subjective assessment. It is linked to a number of factors, including the patient's emotional, social, and cultural beliefs, past experiences, and expectations for the future (1, 2).

It demonstrates how well the care provided meets the expectations of the patients. Patients want to compare their expectations to their previous experiences as well as the actual outcomes. The patient may get disappointed if their expectations are not met by the real circumstances. As a result, the consistency between the patient's anticipation and what they actually perceive or experience determines patient satisfaction (3, 4).

Patient satisfaction is an important aspect of care quality. Patients are more satisfied when the quality of service is improved (5, 6). Furthermore, patient loyalty is mostly determined by the quality of service and client pleasure (7). Loyal clients are more likely to return for service, and they may also promote it to their relatives, friends, and other service seekers (8).

Parental satisfaction is a quality measurement that is being more commonly used as a method to get improved the child's anesthetic or surgical care (9). Many pediatric patients are unable to evaluate and express their happiness because they are too young. As a result, parental satisfaction with anesthetic care is utilized as a proxy for child satisfaction (10).

In many pediatric healthcare facilities, parent participation in healthcare has become the new standard of care. Between parents and direct care providers, effective communication is essential. Parents desire to have a say in how their children's health is managed. They place a premium on communication that is both open and honest (11–13).

Receiving feedback from parents can provide valuable insight into the anesthetic service's quality (14, 15). Service quality is a determining factor for an institution's success and survival since it fosters client loyalty and keeps the organization competitive (16).

Having enough data on patient satisfaction is thought to help enhance and comprehend its strengths, as well as to identify areas where performance is lacking, and it allows for the improvement or alteration of identified gaps (2, 5, 17). Furthermore, assessing patient satisfaction with the care they receive is an important tool that is increasingly being employed in payment models and pay-for-performance schemes. Payment for anesthetic services will most likely be contingent on patient satisfaction in the future (5). This study was aimed to assess the level of parental satisfaction toward their child's anesthesia services and to determine factors that influence satisfaction at a referral hospital in Ethiopia.

## Methods

### Study Design and Period

An institution-based cross sectional study was conducted on 238 parents whose children underwent surgery at a comprehensive specialized referral hospital in Ethiopia, from March 5 to June 28, 2021 to determine the level of parental satisfaction and associated factors about their child's anesthesia service. This paper was registered in research registry with unique identifying number (UIN) of 7,500.

The ethical issue was approved, and the ethical review committee of the college of medicine and health sciences issued an ethical clearance letter. Parents of children who underwent elective or emergency procedures in the study period of time were included. Parents who refused to give consent, absent during data collection period and those with communication barrier were excluded from the study.

### Operational Definition

Parents: refers any care giver, guardian, or attendant who attended the pediatric patient during interview (18).

Pediatrics: age between 0 and 18 years (19).

Parental satisfaction: Parents who scored 45 or more points out of 75 were considered satisfied, while those who scored <45 were regarded unsatisfied (20).

### Sample Size Determination

The sample size was calculated using a single population proportion formula. Sample size was determined with a study conducted in Lagos University Teaching Hospital, south west Nigeria, which reported that the overall parents' satisfaction with pediatric perioperative anesthesia care was 82% (21).

The size of the study participants was calculated using a 95% level of confidence and a 5% margin of error.


(1) $$\begin{array}{r} \text{n}=\frac{{p\left(1-p\right)\left(za/2\right)}^{2}}{d^{2}} \end{array}$$

where *n* is the sample size, *p* is the proportion, *q* = 1 – *p, d* is the desired degree of precision, and *z* is the standard normal value at the appropriate level of confidence.


(2) $$\begin{array}{r} \text{n}=\frac{0.82\times \left[1-0.82\right]{\left[1.96\right]}^{2}}{{\left[0.05\right]}^{2}} \end{array}$$

$$\begin{array}{r} \text{n}=227 \end{array}$$

The total number of parents that participated in the study was 238 after the non-response rate of 5% was included in.

### Data Collection Procedure

Parents were interviewed using a pre-tested and standardized questionnaire. Data were extrapolated from the patients' chart and anesthetic record sheets that included diagnosis, coexisting medical illness, and urgency of the procedure, type of procedure, ASA physical status, and duration of anesthesia. Parents were asked for general demographic information regarding both themselves and their child. Training was given to data collectors.

Modified Yale Preoperative Anxiety Scale (m-YPAS) (22) and State-Trait Anxiety Inventory (STAI) (23) were used to assess both child's and parent's preoperative anxiety, respectively.

A valid and accurate assessment method, the Pediatric Anesthesia Parent Satisfaction (PAPS) questionnaire, was utilized to assess parental satisfaction with anesthesia services. A 5-point Likert scale was used to evaluate the parent satisfaction. Based on the demarcation threshold calculation, a five-point Likert scale was adopted from the PAPS questionnaire and dichotomized as satisfied or unsatisfied (20).

Because each item had a 5-point Likert scale ranging from 1 to 5, the scores for each dimension were determined by adding the responses to all of the items in that dimension. Using the demarcation threshold methodology, parents' overall and component-wise levels of satisfaction were divided into two categories: satisfied and unsatisfied. The formula for determining the demarcation threshold is ([total highest score – total lowest score]/2 + total lowest score). Patients who scored <45 out of 75 were judged unhappy, while those who scored 45 or higher were considered satisfied. Parents were given the PAPS questionnaire, which was graded on a five-point Likert scale from strongly agree to strongly disagree.

### Data Quality Management

The lead investigator offered training to data collectors. A pre-test on 10% of parents who were not involved in the main study was undertaken to assure data quality. The questionnaire for the main study received necessary modifications. Important information regarding assessment tools and the questionnaire was provided to parents. The collected data were reviewed by the lead investigator for completeness and accuracy.

### Data Processing and Analysis

Epi-Data version 4.6 was used to enter data, which was then exported to SPSS version 20 for analysis. Socio-demographic variables of the parent and child as well as descriptive part of the survey scores of parent satisfaction, child and parent anxiety were analyzed and presented in tables, graphs, and narrations.

All normally distributed data were presented by means and standard deviation. Variables with a *p*-value of 0.02 in binary logistic regression were transferred to multivariable logistic regression, and variables with a *p*-value of 0.05 with a 95% confidence interval (*CI*) were deemed predictor variables for parental satisfaction with their child's anesthetic service.

## Results

### Socio-Demographic and Related Characteristics of Parents and Children

Two hundred thirty eight (238) parents whose children had surgery and anesthesia were included in the study, with a 100% response rate. Out of the total parents, 121 (50.8%) were women. The participants had a mean age of 36.3 years (±9.28). About ninety three (39.1%) parents had no formal education. Hundred and twenty six (52.9%) parents were from urban areas (Table 1).

**Table 1:** Socio-demographic characteristics of parents related with parental satisfaction toward their child's anesthesia service, *N* = 238, 2021.

**Characteristics**	**Frequency (*n*)**	**Percentage (%)**
**Sex of the parent**		
Female	121	50.8
Male	117	49.2
**Age of the parent**		
<30	43	18.1
30–40	85	35.7
41–50	80	33.6
Above 50	30	12.6
**Place of residency**		
Urban	126	52.9
Rural	112	47.1
**Marital status**		
Single	63	26.5
Married	168	70.6
Divorced	6	2.5
Widowed	1	0.4
**Educational level**		
No formal education	93	39.1
Primary education	43	18
High school and above	102	42.9
**Occupation**		
Farmer	90	37.8
House wife	25	10.5
Merchant	40	16.8
Employee	41	17.2
Others	42	17.7
**Monthly income in ETB**		
≤150	45	18.9
151–600	43	18.1
601–1,200	29	12.2
1,201–2,500	50	21
>2,501	71	29.8
**Religion**		
Orthodox Christian	211	88.7
Muslim	26	10.9
Protestant	1	0.4
**Language**		
Amharic	234	98.3
Tigrigna	4	1.7

*ETB, Ethiopian Birr*.

The mean age of children was 8.6 years (±5.3). Majority of children, 154 (64.7%), were male. About 50.8% of children were at school. Most of the children, 191 (80.3%), were American society of anesthesiologist (ASA) class I. About 222 (93.3%) children had operation under general anesthesia. More than half of the procedures, 128 (53.8%), were emergency operations. Only twelve (5%) were oncologic procedures. Fifty four (22.7%) procedures were general surgery. Only twenty (8.4%) children had previous anesthetic and surgical history (Table 2).

**Table 2:** Socio-demographic and related characteristics of children, *N* = 238, 2021.

**Characteristics**	**Frequency**	**Percentage**
**Age (year)**		
<1	31	13
1–4	73	15.1
5–10	36	30.7
11–18	98	41.2
**Gender**		
Male	154	64.7
Female	84	35.3
**Child educational status**		
Illiterate	117	49.2
School	121	50.8
**ASA status**		
ASA I	191	80.3
ASA II	47	19.7
**Urgency of procedure**		
Elective	110	46.2
Emergency	128	53.8
**Types of procedure**		
General surgery	54	22.7
Orthopedics surgery	50	21.0
Ophthalmic surgery	48	20.2
ENT surgery	33	16.4
Neuro surgery	14	5.9
Other	39	13.9
**Type of anesthesia**		
General anesthesia	222	93.3
Regional anesthesia	16	6.7
**Duration of anesthesia**		
<90 min	100	42
>90 min	138	58
**Previous anesthesia history**		
Yes	20	8.4
No	218	91.6

*ASA, American Society of Anaesthesiologist; ENT, Ear, nose and throat*.

### Level of Parental Satisfaction Toward Their Child'S Anesthesia Service

The overall proportion of parental satisfaction about their child's anesthesia service was 77.73% (95% *CI*: 72.3, 82.4%). Majority of respondents, 221 (92.9%), were willing to recommend this anesthesia team to others in their families. About 148 (63.6%) respondents were satisfied with the response of anesthesia team to all their questions prior to their child's surgery. Majority of parents (73%) were satisfied on the amount of information provided by the anesthesia team (Table 3).

**Table 3:** Level of parental satisfaction toward their child's anesthesia service, *N* = 238, 2021.

**Items**	**Satisfied (*n*, %)**	**Dissatisfied (*n*, %)**
Before anesthesia; The anesthesia team answered all my questions Prior to my child's surgery.	148 (62.18%)	90 (37.82%)
Before anesthesia; I was satisfied with the amount of information provided by the anesthesia team.	175 (73.53%)	63 (26.47 %)
Before anesthesia; The information given to me by the anesthesia team was understandable	201 (84.45%)	37 (15.55%)
Before and After Anesthesia; The anesthesia team explained to me how my child might feel physically and emotionally.	150 (63.03%)	88 (36.97%)
Before and After Anesthesia; I was satisfied with the way my child fell asleep and woke up from anesthesia.	78 (32.77%)	160 (67.23%)
After Anesthesia; I felt my child's pain was well-controlled.	102 (42.9%)	136 (57.1%)
After anesthesia; I felt my child's nausea and vomiting was well-controlled.	97 (40.8%)	141 (59.2%)
Hospital team (staff) we met for my child's surgery behaved professionally & respectfully.	184 (77.3%)	54 (22.7%)
My child received the highest quality care during this surgical experience by the hospital team.	154 (64.7%)	84 (35.3%)
The Anesthesia team behaved professionally and respectfully toward my child.	169 (71%)	69 (29%)
My child's privacy was respected at all times by the anesthesia team.	190 (79.8%)	48 (20.2%)
The anesthesia team paid attention to my concerns regarding my child's care.	152 (63.9%)	86 (36.1%)
Anesthesia team; I was satisfied with the care my child received by the anesthesia team.	194 (81.5%)	44 (18.5%)
I know who the anaesthesiologist was and his/her role in my child's care.	169 (71%)	69 (29%)
I would recommend this anesthesia team to others in my family.	214 (89.9%)	24 (10.1%)

Hundred and ninety (79.8%) parents were satisfied with their child's privacy respect at all times by the anesthesia team, whereas hundred and fifty two (63.9%) parents were satisfied with attention and concerns of the anesthesia team to their child's care (Table 3).

On the other hand, about 136 (57.1%) parents were not satisfied with their child's pain was well-controlled. More than half (59.2%) of parents were not feel their child's nausea and vomiting after anesthesia was well-controlled. The majority of parents (67.22%) were unhappy with how their child fell asleep and awoke after anesthesia (Table 3).

### Bi-Variable Analysis

In the bi-variate logistic regression, variables like sex of the parent, parental anxiety, sedative premedication, place of residency, parental occupation, number of children in family, parental education status, child anxiety, house hold income, duration of anesthesia, and urgency of surgery had a *p*-value of < 0.2 and considered to be factors associated with parental satisfaction about their child's anesthesia service (Table 4).

**Table 4:** Bi-variable logistic regression analysis of factors associated with parental satisfaction toward their child's anesthesia service, *N* = 238, 2021.

**Variables**	**Level of satisfaction**		**Odds ratio**
	**Satisfied (*n*, %)**	**Dissatisfied (*n*, %)**	**COR (95% CI)**
**Parental anxiety**			
Yes	106 (69.3)	47 (30.7%)	1
No	76 (89.4%)	9 (10.6%)	3.7 (1.731, 8.10)
**Sex of parents**			
Female	84 (69.4%)	37 (30.6%)	1
Male	98 (83.8%)	19 (16.2%)	2.272 (1.216, 4.26)
**Sedative premedication**			
Yes	71 (83.5)	14 (16.5%)	1.708 (0.881, 3.34)
No	114 (74.5%)	39 (25.5%)	1
**Residency**			
Urban	105 (83.3%)	21 (16.7%)	2.27 (1.228, 4.2)
Rural	77 (68.8%)	35 (31.2%)	1
**Parental occupation**			
Farmer	70 (77.8%)	20 (22.2%)	1
House wife	15 (60.0%)	10 (40.0%)	2.333 (0.91, 5.985)
Employee	34 (82.9%)	7 (17.1%)	3.23 (1.035, 10.1)
Merchant	37 (92.5%)	3 (7.5%)	8.22 (1.982, 4.114)
Other	26 (61.9%)	16 (38.1%)	1.08 (0.393, 2.986)
**Child anxiety**			
Yes	119 (73%)	44 (27.0%)	1
No	66 (88.0%)	9 (12.0%)	2.71 (1.246, 5.901)
**Income (ETB)**			
≤150	13 (28.9%)	32 (71.1%)	1
151–600	11 (25.6%)	32 (74.4%)	0.715 (0.290, 1.7)
601–1,200	4 (13.8%)	25 (86.2%)	1.535 (0.45, 5)
1,201–2,500	13 (26.5%)	36 (73.5%)	1.5 (0.459, 5.1)
>2,501	57 (80.3%)	14 (19.7%)	0.68 (0.287,1.6)
**Urgency of surgery**			
Elective	83 (75.5%)	27 (24.5%)	1.162 (0.635, 2.1)
Emergency	28 (21.9%)	100 (78.1%)	1
**Duration of anesthesia**			
<90 min	80 (80.0%)	20 (20%)	0.736 (0.395, 1.371)
>90 min	35 (25.4%)	103 (74.6%)	1
**Educational status of parent**			
No formal education	68 (73.1%)	25 (26.9%)	1
Primary education	28 (65.1%)	15 (34.9%)	0.542 (0.266, 1.106)
High school and above	86 (84.3%)	16 (15.7%)	0.349 (0.155, 0.783)
**Number of children in family**			
1	27 (84.4%)	5 (15.6%)	1
2–4	102 (78.5%)	28 (21.5%)	2.062 (0.701, 6.063)
>5	55 (72.4%)	21 (27.6%)	1.391 (0.723, 2.675)

*COR, crude odds ratio; CI, Confidence interval; 1, reference*.

### Multi-Variable Analysis

In multivariable logistic analysis, variables such as sex of parent, parental anxiety, sedative premedication, place of residency, and parental occupation had a *p*-value of < 0.05 and were considered as significant predictors of parental satisfaction toward their child's anesthetic service (Table 5).

**Table 5:** Multi-variable logistic regression analysis of factors associated with parental satisfaction toward their child's anesthesia service, *N* = 238, 2021.

**Variables**	**Level of satisfaction**		**Odds ratio**		
	**Satisfied** **(*n*, %)**	**Dissatisfied** **(*n*, %)**	**COR (95% CI)**	**AOR (95% CI)**	***P***
**Parental anxiety**					
Yes	106 (69.3)	47 (30.7%)	1	1	
No	76 (89.4%)	9 (10.6%)	3.7 (1.731, 8.100)	3.45 (1.303, 9.15)	0.013^*^
**Sex of parents**					
Female	84 (69.4%)	37 (30.6%)	1	1	
Male	98 (83.8%)	19 (16.2%)	2.272 (1.216, 4.2)	2.578 (1.147, 5.79)	0.022^*^
**Premedication**					
Yes	71 (83.5)	14 (16.5%)			
No	114 (74.5%)	39 (25.5%)	1.708 (0.881, 3.3)	2.3 (1.037, 503)	0.038^*^
**Residency**					
Urban	105 (83.3%)	21 (16.7%)	2.27 (1.228, 4.2)	2.904 (1.093, 7.71)	0.033^*^
Rural	77 (68.8%)	35 (31.2%)	1	1	
**Occupation**					
Farmer	70 (77.8%)	20 (22.2)	1	1	
House wife	15 (60.0%)	10 (40%)	2.333 (0.910, 5.985)	1.021 (0.281, 3.71)	0.9750
Other	26 (61.9%)	16 (38.1)	1.08 (0.393, 2.986)	1.09 (0.360, 3.32)	0.87
Merchant	37 (92.5%)	3 (7.5%)	8.22 (1.98234.114)	6.3 (1.45, 28)	0.014^*^
Employee	34 (82.9%)	7 (17.1%)	3.23 (1.035, 10.1)	3.7 (0.797, 17.5)	0.094

*1, Reference*.

* *Significant; AOR, Adjusted Odds Ratio; COR, Crude Odds Ratio*.

### Factors Associated With Level of Parental Satisfaction

Multi-variable logistic regression analysis showed that urban residents were 2.9 times (*CI*: 1.093, 7.71) more likely to be satisfied than rural residents. Male parents were 2.57 times more likely than female parents to be satisfied (*CI*: 1.14, 5.79). Employed parents were 4.09 times more likely than farmer parents to be satisfied (*CI*: 1.043, 16.05). Non-anxious parents were 3.45 times more likely to be satisfied than anxious parents (*CI*: 1.303, 9.15). Parents whose children received premedication were 2.29 times more likely to be satisfied than parents whose children did not (*CI*: 1.037, 5.03) (Table 5).

## Discussion

This research was conducted to determine the level and associated factors of parental satisfaction with their Child's anesthesia service at a comprehensive specialized referral hospital in Ethiopia.

The overall proportion of parental satisfaction about their child's anesthesia service was 77.7% (95% *CI*: 72.3, 82.4%). Despite the fact that our department had implemented a preoperative assessment 2 days before to surgery, parents' satisfaction with their child's anesthesia service was suboptimal. One possible explanation is a lack of attention and concern paid to the idea that parental happiness is a crucial outcome and quality predictor of healthcare delivery. The level of parental satisfaction in our study was similar with the findings of a study conducted in Nigeria (82%) (21); however, lower than a result reported in India (88%) (24). This is most likely attributable to socio-cultural and income variations among parents, as well as differences in the quality of healthcare services.

In our study, parental satisfaction level was higher compared to other study done in Black Lion Specialized Hospital in Ethiopia (59.8%) (13). This may be related to our hospital's implementation of preoperative examination 2 days before to surgery by senior anesthetists. This practice will allow for a positive parent–anesthetist interaction and a better comprehension of the information provided to parents about anesthesia and surgery. Parents want to have entire knowledge of anesthesia plans, including information about all possible benefits and adverse effects of drugs, thus proper communication between healthcare providers and patients or their families is essential. The preoperative communication between the healthcare providers and the parents, as well as proper information distribution to the family, minimizes parental worry and promotes satisfaction with the care (25).

Non-anxious, male, employed, and urban resident parents and parents of pre-medicated children were associated with parental satisfaction with their child's anesthesia service.

Non-anxious parents were 3.4 times more satisfied with their child's anesthetic service than anxious parents. This finding was consistent with findings from similar research in Turkey and Hong Kong, which found that lower levels of parental worry were linked to better levels of satisfaction (26, 27). Non-anxious parents manage problems by asking questions as they arise and having discussions with doctors who provide quick feedback, psychological support, and anesthetic care instructions for their children, resulting in a high level of satisfaction. Non-anxious parents will be able to communicate effectively with medical specialists and comprehend facts regarding their child's condition and health (26, 27).

The findings of this study demonstrated that male parents were 2.5 times more likely than female parents to be satisfied with their child's overall anesthetic care. This result was in line with the findings of a previous study conducted in Kenya (28). It's likely because fathers pay more attention to their children's outward behavior than mothers do, and hence are better able to predict their children's outward reactions during anesthetic induction. This explanation matched the previous research demonstrating that fathers engage in more active play with their children and are more inclined to agree with their children's conduct judgments (24). Most parents (75%) wanted to know about all potential hazards; however, this was more common among mothers than fathers, which could be due to the fact that men are more prone to express concerns than women, who worry that if they complain, their child would not be well-cared for (19).

Our findings revealed that parents in urban areas were 2.9 times more likely than those in rural areas to be satisfied with their child's anesthetic care. Another study in Ethiopia found that parents in urban areas were more likely to be satisfied than their counterparts in rural areas (13). This is due to the fact that urban residents are aware of their patient autonomy and are capable of asking healthcare practitioners questions regarding their current state, progress, and the intervention plan that will be implemented (29).

Parents of children who received premedication were also 2.2 times more likely to be satisfied than parents of children who did not get premedication. During the preoperative period, anxiety is a significant negative impact for both children and their families. Both the child and the parent experience anxiety when they are separated from one another. Parents might be distressed to see their child is frightened. Premedication with sedatives and detailed information can help to reduce stressful situations and boost parental satisfaction (30).

The pleasure of parents with their child's anesthetic care was also influenced by their own profession. The likelihood of employed parents being satisfied with their child's anesthetic care was four times higher than that of farmer parents. Employed parents were shown to have a higher level of education and lower perioperative anxiety ratings, according to studies (31). Parents with a high level of education will be more satisfied since they will have more information and understanding about their right to know about anesthetic care (26).

## Conclusion and Recommendation

Overall, parent's satisfaction with their child's anesthesia service was promising. Parents who were non-anxious, male, employed, and lived in an urban area and whose child had received sedative premedication had high rates of parental satisfaction with their child's anesthetic care.

Parents from rural areas, as well as worried and female parents, should receive extra care. Preoperative anxiety is reduced when parents are given enough and understandable information. Thus, the provision of comprehensive information on their child's anesthesia care process and psychological or emotional support to parents are necessary to boost their satisfaction.

## Strength and Limitation

The study can be considered a base for future studies as there were few similar studies conducted in Ethiopia.

This was a single-center study and conducted on small participants which may limit its representativeness. The data were obtained solely through self-report measures which may be subjected to participant bias.

## Data Availability Statement

The original contributions presented in the study are included in the article/supplementary material, further inquiries can be directed to the corresponding authors.

## Ethics Statement

The study was approved by the Ethical Committee of College of Medicine and Health Sciences and received an ethical clearance letter with a reference number of No/479/4/202. After a thorough explanation, each study participant signed a written informed consent form. By minimizing personal identifying and locking the completed questionnaires, confidentiality was ensured.

## Author Contributions

BA, AH, AA, and AG contributed to the study's idea and design, data collection, analysis and interpretation, manuscript drafting, and final approval of the manuscript version. All authors contributed to the article and approved the submitted version.

## Conflict of Interest

The authors declare that the research was conducted in the absence of any commercial or financial relationships that could be construed as a potential conflict of interest.

## Publisher's Note

All claims expressed in this article are solely those of the authors and do not necessarily represent those of their affiliated organizations, or those of the publisher, the editors and the reviewers. Any product that may be evaluated in this article, or claim that may be made by its manufacturer, is not guaranteed or endorsed by the publisher.
